# Oral GS-441524 derivatives: Next-generation inhibitors of SARS‐CoV‐2 RNA‐dependent RNA polymerase

**DOI:** 10.3389/fimmu.2022.1015355

**Published:** 2022-12-06

**Authors:** Zhonglei Wang, Liyan Yang, Xian-qing Song

**Affiliations:** ^1^ Key Laboratory of Green Natural Products and Pharmaceutical Intermediates in Colleges and Universities of Shandong Province, School of Chemistry and Chemical Engineering, Qufu Normal University, Qufu, China; ^2^ School of Pharmaceutical Sciences, Tsinghua University, Beijing, China; ^3^ Shandong Provincial Key Laboratory of Laser Polarization and Information Technology, School of Physics and Physical Engineering, Qufu Normal University, Qufu, China; ^4^ General Surgery Department, Ningbo Fourth Hospital, Xiangshan, China

**Keywords:** SARS‐CoV‐2, GS‐441524, remdesivir, oral version, VV116, ATV006, GS-621763, COVID‐19

## Abstract

GS-441524, an RNA‐dependent RNA polymerase (RdRp) inhibitor, is a 1′-CN-substituted adenine C-nucleoside analog with broad-spectrum antiviral activity. However, the low oral bioavailability of GS‐441524 poses a challenge to its anti-SARS-CoV-2 efficacy. Remdesivir, the intravenously administered version (version 1.0) of GS-441524, is the first FDA-approved agent for SARS-CoV-2 treatment. However, clinical trials have presented conflicting evidence on the value of remdesivir in COVID-19. Therefore, oral GS-441524 derivatives (VV116, ATV006, and GS-621763; version 2.0, targeting highly conserved viral RdRp) could be considered as game-changers in treating COVID-19 because oral administration has the potential to maximize clinical benefits, including decreased duration of COVID-19 and reduced post-acute sequelae of SARS-CoV-2 infection, as well as limited side effects such as hepatic accumulation. This review summarizes the current research related to the oral derivatives of GS-441524, and provides important insights into the potential factors underlying the controversial observations regarding the clinical efficacy of remdesivir; overall, it offers an effective launching pad for developing an oral version of GS-441524.

## Introduction

With more than 6.5 million deaths worldwide, the coronavirus disease 2019 (COVID-19) pandemic, first identified in late 2019, continues to be the most extraordinary public health burden in 2022 (1). Severe acute respiratory syndrome coronavirus 2 (SARS-CoV-2) causes a wide range of post-acute infection syndromes, including cognitive impairment, dyspnea, fatigue, headache, and loss of taste or smell (2–5). The scientific community has made significant progress in mitigating the threat of COVID-19 through the discovery and development of myriad vaccines, monoclonal antibodies, traditional medicines, and small‐molecule agents (6–9). However, the efficacy of antibodies or vaccines has been affected by the development of SARS-CoV-2 variants (i.e., their drug resistance), global access, sub-optimal administration routes, and heterogenous responses (10, 11). Briefly, resistant SARS-CoV-2 variants have led to waves of resurgence, exacerbated global anxiety, and challenged global health efforts.

As an acute infectious disease, use of orally bioavailable antivirals at the early stage of SARS-CoV-2 infection would be more beneficial in facilitating early administration to non-hospitalized patients to prevent progression to severe disease; however, antiviral treatment does not provide the best benefits in the late stage in hospitalized patients (12, 13). Therefore, oral antivirals suitable for outpatient treatment are superior to injectable therapies for hospitalized patients. Currently, oral antivirals are critical as pre- and post-exposure prophylaxis. Notably, several orally bioavailable anti-SARS-CoV-2 agents have been approved. Among these, Paxlovid (nirmatrelvir plus ritonavir) from Pfizer and molnupiravir (MK-4482, EIDD‐2801) from Merck can effectively reduce the risk of severe COVID-19 or death in patients with mild-to-moderate COVID-19 (14–16). Although these results are appealing, some concerns remain. First, despite a high cure rate with initial therapy, patients treated with Paxlovid and molnupiravir have experienced rebound COVID-19 infections (17, 18). Second, mutations are challenging the efficiency of these small‐molecule antivirals. Third, commercialized antivirals remain expensive (around US $530 for each 5-day course of Paxlovid), particularly, for low and middle-income countries (19, 20). Fourth, the widening use of antivirals can increase the development of drug resistance. Fifth, molnupiravir has mutagenic potential in human cells (21, 22). Based on these concerns, efforts are needed to achieve the desired clinical effects (e.g., tissue-specific localization and enhanced oral bioavailability) of antivirals.

RNA‐dependent RNA polymerase (RdRp) is essential in viral RNA synthesis (23–25). Both remdesivir and monapivir are FDA-approved SARS-CoV-2 inhibitors that target RdRp. Notably, remdesivir exhibited conflicting impact in clinical trials (26); rebound of COVID-19 infection and mutagenicity may occur in patients receiving molnupiravir (27). In such cases, oral GS-441524 derivatives, with enhanced bioavailability and sufficient safety profile, may be another alternative to consider.

GS-441524, a potent RdRp inhibitor, is a 1′-CN-substituted adenine C-nucleoside ribose analogue that demonstrates *in vitro* broad‐spectrum activity against various viruses, including SARS‐CoV (half-maximum effective concentration [EC_50_] = 0.18 μM), Middle East respiratory syndrome coronavirus (EC_50_ = 0.86 μM), and feline infectious peritonitis virus (EC_50_ = 0.78 μM), SARS‐CoV‐2 (EC_50_ = 0.48 μM) (28–31). Cellular uptake dependents on membrane-bound transporters, and GS-441524 is hydrophilic, resulting in a limited ability to transmembrane by diffusion (32). Notably, GS-441524 displayed low oral bioavailability in cynomolgus monkeys (*F* < 8.0%), in rats (*F* = 16%) and humans (*F* = 13%) (33). Other detailed pharmacokinetic properties (such as maximum plasma concentration, terminal half-life, and oral bioavailability) of GS-441524 are shown in **Table 1**. Also, GS-441524 is stable *in vitro* in liver microsomes, cytoplasm, and hepatocytes across studied species (including rats, monkeys, dogs, and humans) (37). Further, clinical trials have shown that GS-441524 is the major circulating metabolite of remdesivir after IV administration (44). GS-441524 displays a good safety profile without serious adverse effects (45). Therefore, GS-441524 and its prodrugs or analogues provide an excellent option for oral SARS-CoV-2 drug design.

**Table 1:** Pharmacokinetic properties of GS-441524, VV116, and related compounds.

Compound	*In vivo*model	Route of administration	Dose(mg/kg)	Pharmacokinetic parameters				
				AUC_last_	T_1/2_ (h)	T_max_ (h)	C_max_	*F* (%)
GS-441524	rat (34, 35)	i.v.	5	11.0 (μM*h)	1.2		10.7 μM	
		p.o.	25	11.7 (μM*h)	1.4	1.0	3.4 μM	21.7
		i.v.	30	591.9 (μg/mL*h)	4.8	0.1	163.6 (μg/mL)	
		p.o.	30	28.7 (μg/mL*h)	20.6	0.9	2.7 (μg/mL)	4.8
	CD-1 mice (36)	i.v.	5	14.8 (μM*h)	2.5	0.08	11.6 μM	
		p.o.	10	16.8 (μM*h)	2.9	1.0	3.3 μM	57.0
	mic (37)	p.o.	10	<2540 (ng/mL*h)	3.9	1.5	582 (ng/mL)	39.0
	rat (37)	p.o.	10	<2170 (ng/mL*h)	3.4	3.8	193 (ng/mL)	33.0
			30	<4220 (ng/mL*h)	3.4	3.8	326 (ng/mL)	21.0
			100	<9560 (ng/mL*h)	4.2	2.3	825 (ng/mL)	15.0
	monkey (37)	p.o.	5	<734 (ng/mL*h)	7.7	2.0	59.4 (ng/mL)	8.3
	dog (37)	p.o.	5	< 19000 (ng/mL*h)	4.1	0.3	6010 (ng/mL)	85.0
	dog (38)	i.v.	2	26.6 (μM*h)	3.6	0.5	1897 (ng/mL)	
		p.o. (solution)	5	61.5 (μM*h)	4.0	0.5	5060 (ng/mL)	92.0
		p.o. (capsule)	6.5	65.6 (μM*h)	3.4	1.0	4580 (ng/mL)	76.0
	patients (39)	p.o. (normal renal function)	100 mg	1582 (ng/mL*h)	25.5	0.5	102 (ng/mL)	–
				1905 (ng/mL*h)	13.3	0.5	157 (ng/mL)	–
		p.o. (impaired renal function)	100 mg	7728 (ng/mL*h)	43.7	12	356 (ng/mL)	–
				11060 (ng/mL*h)	26.2	0.5	563 (ng/mL)	–
		p.o. (impaired renal function receiving CRRT)	100 mg	9203 (ng/mL*h)	48.6	0.5	436 (ng/mL)	–
				8213 (ng/mL*h)	37.8	0.5	421 (ng/mL)	–
		p.o. (impaired renal function receiving IHD)	100 mg	25615 (ng/mL*h)	70.4	2.5	1653 (ng/mL)	–
				26950 (ng/mL*h)	–	–	1280 (ng/mL)	–
				9785 (ng/mL*h)	82.5	0.5	706 (ng/mL)	–
ATV006	rat (35)	i.v.	5	5.6 (μM*h)	1.5		8.7 μM	
		p.o.	25	22.8 (μM*h)	1.2	0.5	8.2 μM	81.5
	monkey (35)	i.v.	5	20.5 (μM*h)	1.8	0.08	12.8 μM	
		p.o.	10	12.2 (μM*h)	4.1	1.5	3.7 μM	30.1
GS-621763	ferrets (40)	p.o.	30	80.8 (μM*h)	2.7	4.0	15.8 μM	115.0
GS-621763 ·HBr	mice (31)	i.v.	25	3603 (ng/mL*h)	2.5	–	–	
		p.o.	50	7112 (ng/mL*h)	2.7	0.3	3613 (ng/mL)	98.7
X1	rat (41)	i.v.	2	1724 (ng/mL*h)	6.7		1630 (ng/mL)	
		p.o.	10	1869 (ng/mL*h)	4.5	2.0	246 (ng/mL)	21.7
X2	rat (41)	i.v.	2	1611 (ng/mL*h)	5.0		1570 (ng/mL)	
		p.o.	10	2556 (ng/mL*h)	4.1	2.0	246 (ng/mL)	32.6
	monkey (41)	i.v.	5	6814 (ng/mL*h)	1.3		3322 (ng/mL)	
		p.o.	10	1939 (ng/mL*h)	2.0	1.0	941 (ng/mL)	14.2
X2-H	mice (31)	i.v.	25	7981 (ng/mL*h)	4.8		–	
		p.o.	50	13817 (ng/mL*h)	2.5	0.4	6677 (ng/mL)	86.6
X3	rat (41)	i.v.	2	1915 (ng/mL*h)	6.7		2520 (ng/mL)	
		p.o.	10	4455 (ng/mL*h)	4.0	0.3	801 (ng/mL)	46.5
	monkey (41)	i.v.	5	6780 (ng/mL*h)	1.4		7766 (ng/mL)	
		p.o.	10	2497 (ng/mL*h)	2.3	1.0	1064 (ng/mL)	18.4
X3-H	mice (31)	i.v.	25	10174 (ng/mL*h)	0.8		–	
		p.o.	50	14565 (ng/mL*h)	1.1	0.3	7879 (ng/mL)	71.6
X6	rat (41)	i.v.	2	1820 (ng/mL*h)	1.3		1693 (ng/mL)	
		p.o.	10	4539 (ng/mL*h)	2.0	0.3	1370 (ng/mL)	49.9
VV116	rat (41)	i.v.	10	4582 (ng/mL*h)	1.4		–	
		p.o.	10	3960 (ng/mL*h)	1.4	1.0	785 (ng/mL)	86.4
			30	10883 (ng/mL*h)	2.1	0.8	2068 (ng/mL)	79.2
			90	32807 (ng/mL*h)	3.6	0.8	6040 (ng/mL)	79.6
		p.o. (multiple)	7*30	7743 (ng/mL*h)	3.9	2.0	1740 (ng/mL)	–
	dogs (41)	i.v.	10	15835 (ng/mL*h)	3.9		–	
		p.o.	10	13845 (ng/mL*h)	4.5	1.0	3218 (ng/mL)	87.4
			20	32206 (ng/mL*h)	4.2	1.1	6633 (ng/mL)	101.7
			40	63289 (ng/mL*h)	4.3	1.1	11058 (ng/mL)	99.9
		p.o. (multiple)	7*20	27372 (ng/mL*h)	4.3	1.2	6317 (ng/mL)	–
	ICR mice (42)	i.v.	5	2341 (ng/mL*h)	1.6		2995 (ng/mL)	
		p.o.	25	12868 (ng/mL*h)	4.5	0.3	6500 (ng/mL)	110.2
	Balb/c mice (42)	p.o.	25	11461 (ng/mL*h)	2.3	0.4	5360 (ng/mL)	-
			50	24594 (ng/mL*h)	3.3	0.4	11617 (ng/mL)	-
			100	47799 (ng/mL*h)	4.3	0.4	24017 (ng/mL)	-
	Rat (42)	i.v.	5	1774 (ng/mL*h)	0.7		1923 (ng/mL)	
		p.o.	30	9259 (ng/mL*h)	5.2	0.3	2710 (ng/mL)	87.0
	healthy subjects (43)	p.o.	20 mg	744 (ng/mL*h)	4.8	1.0	165 (ng/mL)	-
			200 mg	6631 (ng/mL*h)	5.5	1.0	1096 (ng/mL)	-
			400 mg	12759 (ng/mL*h)	6.2	1.5	1898 (ng/mL)	-
			800 mg	25886 (ng/mL*h)	6.8	2.5	2796 (ng/mL)	-
			1200 mg	28057 (ng/mL*h)	7.0	2.0	3086 (ng/mL)	-
		p.o. (multiple)	1*200 mg	4610 (ng/mL*h)	4.7	1.5	858 (ng/mL)	-
			6*200 mg	9384 (ng/mL*h)	7.6	1.0	1131 (ng/mL)	-
			1*400 mg	10351 (ng/mL*h)	4.9	1.5	1968 (ng/mL)	-
			6*400 mg	20774 (ng/mL*h)	8.1	1.0	2304 (ng/mL)	-
			1*600 mg	12871 (ng/mL*h)	5.4	1.5	2418 (ng/mL)	-
			6*400 mg	25077 (ng/mL*h)	7.9	1.5	2842 (ng/mL)	-
		p.o.	400 mg (Fasting)	10443 (ng/mL*h)	5.7	1.5	1523 (ng/mL)	-
			400 mg (Standard meal)	12405 (ng/mL*h)	5.3	3.0	1583 (ng/mL)	-
			400 mg (High-fat meal)	13107 (ng/mL*h)	5.5	2.5	1602 (ng/mL)	-
VV116-H	rat (42)	i.v.	5	1689 (ng/mL*h)	0.7		1997 (ng/mL)	
		p.o.	30	7664 (ng/mL*h)	4.6	1.0	2060 (ng/mL)	75.6

t_1/2_, terminal half-life; C_max_, maximum plasma concentration; T_max_, time to reach C_max_; AUC_last_, area under the concentration−time curve from the time of dosing to the last quantifiable time point; i.v., intravenous administration; p.o., per os; CRRT, continuous renal replacement therapy; IHD, intermittent hemodialysis.

**Figure 1:**
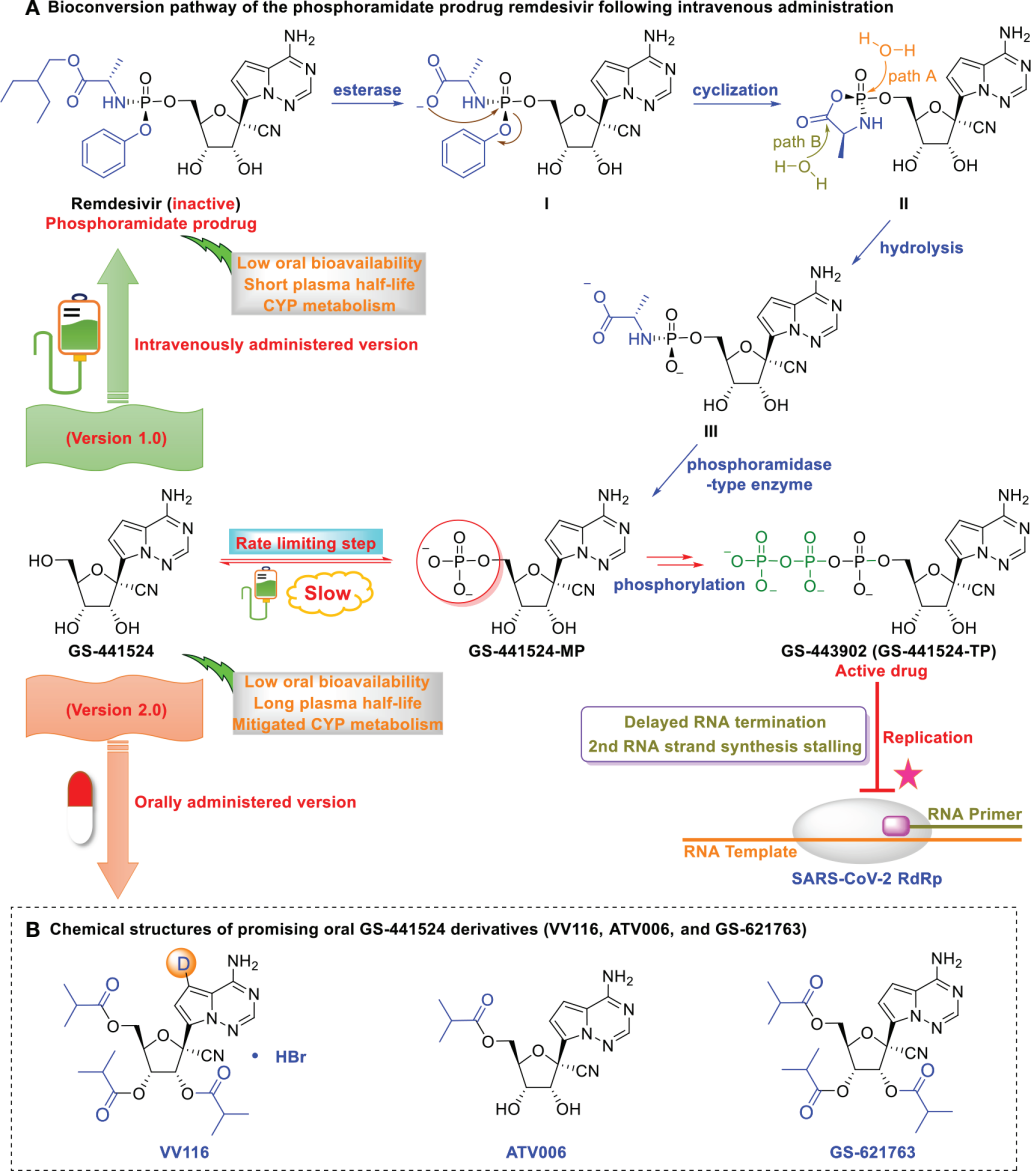
Optimization of GS-441524 for intravenous remdesivir and the oral version, VV116. **(A)** Bioconversion pathway of the phosphoramidate prodrug remdesivir following intravenous administration. Low oral bioavailability renders GS-441524 unsuitable as an oral drug. The first phosphorylation is the rate-limiting step of GS-441524, which renders it less efficient as an intravenous drug. GS‐441524-based lead optimization resulted in the development of the more potent intravenously administered remdesivir (version 1.0). Following intravenous administration, remdesivir distributes to the lung, where it is rapidly converted into its monophosphate metabolite and efficiently anabolized to the bioactive triphosphate form (GS‐443902). However, human microsomal hepatic cytochrome P450 (CYP)-mediated metabolism in the liver and the short plasma half-life of remdesivir renders it unsuitable as an oral drug. **(B)** Chemical structures of promising oral GS-441524 derivatives (VV116, ATV006, and GS-621763). GS-441524 does not undergo human microsomal hepatic cytochrome P450 (CYPs 1A1, 1A2, 3A4, 3A5, 2B6, 2C8, 2C9, 2C19, and 2D6) metabolism and exhibits a long plasma half-life. Further optimization of GS‐441524 has resulted in the development of promising orally administered VV116, ATV006, and GS-621763 (version 2.0).

However, the low oral bioavailability of GS‐441524 hinders its promising anti-SARS-CoV-2 efficacy (33, 34, 46, 47). As depicted in **Figure 1**, further optimization of GS‐441524 has resulted in the development of the more potent intravenously administered remdesivir (version 1.0 of GS-441524) and orally administered GS-441524 derivatives (version 2.0 of GS-441524). This review summarizes the potential factors underlying controversial observations regarding the clinical efficacy of remdesivir, the intravenously administered version (version 1.0) of GS-441524 and the current research related to the oral versions of GS-441524.

## Inhibitors of SARS-CoV-2 RNA-dependent RNA polymerase

**Figure 2:**
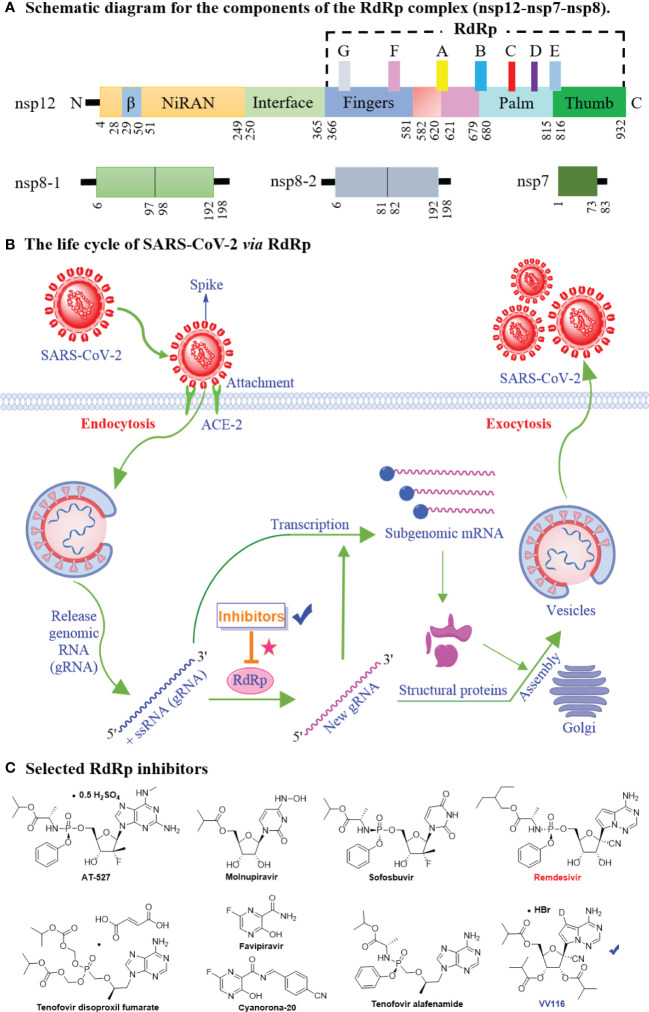
The mechanistic diagram of SARS-CoV-2 RNA-dependent RNA polymerase. **(A)** The schematic diagram for the components of the RdRp complex, containing nsp12, nsp8 and nsp7. The polymerase motif (A to G) and the β hairpin are highlighted. **(B)** The life cycle of SARS-CoV-2 *via* RdRp. The life cycle of SARS-CoV-2 involves sequential viral entry into the target cell, uncoating, replication and transcription, protein synthesis, and viral assembly and release. The RdRp is involved in SARS-CoV-2 genomic and subgenomic mRNA synthesis. **(C)** Chemical structures of selected SARS-CoV-2 RdRp inhibitors **(**AT-527, Molnupiravir, Sofosbuvir, Tenofovir disoproxil fumarate, Favipiravir, Cyanorona-20, Tenofovir alafenamide, Remdesivir, and VV116**)** for COVID-19 treatment.

The SARS-CoV-2 genome encodes 29 proteins, including 4 structural proteins, 16 non-structural proteins (replicase proteins; nsp1-nsp16), and 9 accessory proteins (48, 49). These 29 proteins participate in sequential viral adsorption, entry into the target cell, uncoating, replication and transcription, protein synthesis, SARS-CoV-2 assembly and release (50, 51). Among these, nsp constitutes the viral replication and transcription complex (RTC), which plays an essential role in the synthesis of (-)-strand template, (+)-strand genomic RNA and subgenomic mRNAs (52, 53). Nsp12 (RdRp), the core component of RTC, is one of the most conserved catalytic subunits responsible for RNA synthesis (54). In addition, the co-factors nsp7 and nsp8 enhance the enzymatic activity of nsp12 to catalyze viral RNA synthesis (55). Thus, the core polymerase complex nsp12-nsp7-nsp8 could be called the minimal core component of RNA synthesis (56). A schematic diagram of the core RdRp complex shows in **Figure 2A**. nsp12 contains a right-hand C-terminal RdRp domain with a catalytic cavity (the finger, palm, and thumb subdomains), an N-terminal NiRAN domain (including a novel β-hairpin domain), and an interface domain. In addition, seven conserved motifs (A–G) that mediat template-directed RNA synthesis have been identified in the core RdRp domain (57–59).

Blocking initial SARS-CoV-2 attachment or entry into the target cell and preventing viral replication by suppressing gene transcription are two optimal therapeutic strategies for drug design (**Figure 2B**) (60, 61). That is, RdRp is a key target for antiviral inhibitors. The model of remdesivir (the first approved RdRp inhibitor to treat SARS-CoV-2) binding to nsp12 indicates that remdesivir covalently binds to the 1+ position of the template chain at the central channel, thereby terminating chain extension (62, 63). Based on structural similarities (e.g., remdesivir, molnupiravir, sofosbuvir, and AT-527), nucleoside analogs (**Figure 2C**) are suggested optimal candidates in the search for agents against SARS-CoV-2 (64–70). In brief, the high-resolution crystal structure of SARS-CoV-2 RdRp (PDB ID: 7BTF) has been deciphered, which provides a rational basis for drug design. RdRp plays an essential role in viral RNA synthesis; it is an excellent target in the development of anti-SARS-CoV-2 drugs due to high sequence and structural conservation.

To date, remdesivir, molnupiravir, and paxlovid have been approved for COVID-19 treatment (71). Remdesivir, the first intravenously administered drug targeting RdRp for SARS-CoV-2 treatment, has yielded contradictory clinical results (26). Concerns over renal toxicity, liver injury, and cardiac safety challenge the safety of remdesivir (72–75). Molnupiravir, developed by Merck and Ridgeback Biotherapeutics, is the first approved oral anti-SARS-CoV-2 therapy, which also targets RdRp (15). Concerningly, molnupiravir can induce mutations in mammalian cells and drive new variants (76–78). The use of molnupiravir has been cautioned by the World Health Organization (79). Paxlovid, a main protease inhibitor, is recognized as having a reasonable safety profile (80–82). However, later studies showed that symptoms might reappear after paxlovid treatment, and are often more severe than the initial bout (83). Consequently, there is still a need for safe and effective oral agents. In such cases, oral GS-441524 derivatives (oral version of remdesivir), with sufficient safety and resistance profile, may be more potent SARS-CoV-2 inhibitors against an expansive range of variants.

## Remdesivir: The intravenously administered version of GS-441524

Remdesivir (Veklury^®^, GS-5734), the first FDA-approved intravenously administered drug for SARS-CoV-2 treatment, has exhibited broad-spectrum antiviral activity in studies on *in vitro* models, animal models, and preliminary clinical trials (84–86). Remdesivir, having melting point 89.4-90.4° C and molecular formula C_27_H_35_N_6_O_8_P, has generated widespread interest as a anti-COVID-19 drug (87). However, clinical trials on the value of remdesivir in the treatment of COVID-19 have yielded contradictory results, which have raised several concerns and provided insights.

### Conflicting evidences from clinical trials of remdesivir

To date, several trials have demonstrated promising results with remdesivir. For example, Boglione et al. (88) conducted a single-centre retrospective observational study (time bias and seroprevalence not discussed) in Italy to evaluate the survival, efficacy, and safety of remdesivir treatment in hospitalized patients with COVID-19. In total, 566 participants with similar baseline characteristics underwent randomization:163 patients were assigned to receive remdesivir (200 mg on day 1, followed by 100 mg per day on days 2–5) and 403 patients in the control group were randomly assigned to receive lopinavir/ritonavir, darunavir/cobicistat, or hydroxychloroquine. The COVID-19-related mortality rate was significantly lower in the remdesivir group (4 of 163 participants [2.4%]) than in the control group (100 of 403 [24.8%]) (p < 0.001). Furthermore, the percentage of patients hospitalised in the intensive care unit was lower in the remdesivir group than in the control group (9.8% [16 of 163] *vs.* 17.8% [72 of 403], p = 0.008). Overall, remdesivir-treated patients had a significantly shorter mean hospitalization time than that of the control group (9.5 *vs.* 12.5 days, p < 0.001). No significant adverse drug events were observed in the remdesivir group. Several other studies have produced results consistent with the findings of the Boglione group (89, 90). For example, Gottlieb et al. (89) showed that the percentage of unvaccinated outpatients with COVID-19 who were hospitalized or died was significantly lower in the remdesivir-treated group (200 mg on day 1, followed by 100 mg on days 2 and 3; 2 of 279 participants [0.7%]) than in the placebo group (15 of 283 [5.3%]; 87% lower).

Notably, the anti-SARS-CoV-2 efficacy of remdesivir has also been questioned, as several other studies have found that it adds no value to COVID-19 treatment (91–94). One large-scale, open-label, randomized trial including 11,330 hospitalized adults with COVID-19 (81% aged ≤ 70 years, 38% female) at 405 hospitals in 30 countries was conducted by the World Health Organization to estimate mortality rates. This study indicated that remdesivir did not reduce the mortality rate (remdesivir, 301 of 2743 [11.0%] *vs.* control, 303 of 2708 [11.2%]) or hospitalisation duration (91). Further, Wang et al. (94) conducted a small-scale, double-blind, multicentre, and randomized placebo controlled trial (ClinicalTrials.gov: NCT04257656) to evaluate the efficacy and safety of remdesivir in adults with severe COVID-19 at 10 hospitals in Hubei, China; however, treatment with the drug was not associated with statistically significant benefits. Considering the available clinical evidence, new strategies (such as optimal delivery of the parent nucleoside into systemic circulation) and further investigations may be required to respond to public concerns and to define how remdesivir is best used.

### Practical limitations and countermeasures of remdesivir

Overall, remdesivir has several practical limitations, which need to be addressed to reinforce the value of antivirals. This section addresses the other limitations and concerns regarding remdesivir. First, remdesivir shows strong liver-targeting properties. Remdesivir, the McGuigan (ProTide) prodrug, was initially designed for treating hepatitis C virus infection by overcoming the rate-limiting initial phosphorylation step and sustaining a high concentration of biologically active nucleotide triphosphates (NTP) in the liver (95). However, remdesivir (preferential hepatic extraction) may not be suitable for treating COVID-19 because the lungs are the primary site (type II alveolar cells) of SARS-CoV-2 infection and the most affected organs (96, 97).

Second, remdesivir is not suitable for oral or buccal delivery. Remdesivir shows rapid clearance (plasma half-life < 1 h) owing to its intrinsic hepatic first-pass metabolism and plasma esterase hydrolysis (98, 99). Short exposure is insufficient to achieve the desired anti-SARS-CoV-2 activity. Therefore, remdesivir is highly recommended for only for intravenous therapy in hospitals, which severely limits its application. Although buccal administration is a potential approach to avoid hepatic first-pass metabolism and enzymatic degradation (100), experimental data indicate that cyclodextrin-enabled buccal administration of remdesivir shows only 10% bioavailability (47), suggesting this strategy faces many challenges.

Third, solid preclinical data (overemphasizing low EC_50_) do not reflect the clinical efficacy of remdesivir. For example, Chiu et al. (101) screened a 5676-compound repurposing library of drugs that have passed Phase I clinical trials to identify anti-SARS-CoV-2 candidates; remdesivir alone (EC_50_ values of 5.4 and 1.3 μM in VeroE6-eGFP and Caco-2 cell lines, respectively) was identified as an optimal drug candidate owing to its excellent pharmacodynamic and safety data. However, cell culture studies do not predict the clinical utility of a drug, and it is inappropriate to overrate the clinical efficacy of remdesivir based on *in vitro* cell culture data regarding its efficacy. Thus, additional models and data on remdesivir antiviral therapy are needed.

Finally, the long-term safety of remdesivir remains incompletely examined. Current data indicate that non-uniform distribution of remdesivir could result in high drug accumulation and long-term toxicity; for example, remdesivir potently increases liver transaminases (102) and diminishes the viability of human embryonic stem cells (103) compared to its metabolite, GS-441524. Further, the widespread use of remdesivir in clinical practice raises several concerns regarding the development of drug resistance.

However, the underwhelming clinical performance of remdesivir does not mean that its significance can be disregarded. In contrast, the described limitations provides important insights (such as tissue-specific localization, enhanced oral bioavailability, excellent safety and activity against Omicron variant) to be considered when developing version 2.0 of GS-441524 (targeting highly conserved viral RdRp) for clinical advancement.

## The orally administered versions of GS-441524

Viral RdRp is as a valuable target for treating COVID-19 because it is highly conserved in SARS-CoV-2 variants (104, 105). Use of orally administered versions of GS-441524 at the early stage of the acute infection would be more beneficial in facilitating early administration to non-hospitalized patients to prevent progression to severe disease. Research has shown that oral GS-441524 derivatives (ATV006, VV116, and GS-621763) obtained through individual alterations could be considered game changers for COVID-19 treatment to maximize clinical benefits, including decreased duration of COVID-19 and reduced post-acute sequelae of SARS-CoV-2 infection, as well as limited side effects such as hepatic accumulation. In the following sections, we describe the currently developed orally administered versions of GS-441524.

### ATV006

GS-441524 has its own advantages, further esterification at the 5′-position yielded mono-isobutyrate ester ATV006 with improved oral bioavailability. Xie et al. (36) showed that GS-441524, a promising RdRp inhibitor, demonstrated adequate intracellular conversion into active triphosphate (GS-443902, 42.7–100 nmol/L) in the lungs of CD-1 mice upon oral administration. Furthermore, GS-441524 does not exhibit preferential hepatic metabolism because it is not a substrate for human microsomal hepatic cytochrome P450 (CYPs 1A1, 1A2, 3A4, 3A5, 2B6, 2C8, 2C9, 2C19, and 2D6) (106). Li et al. (34) demonstrated that GS-441524 potently alleviated lung inflammation and injury in AAV-hACE2 mice infected with SARS-CoV-2. However, further development of GS-441524 as an oral drug was hindered by its poor oral bioavailability (*F* = 4.84% in rats) (34).

To develop orally bioavailable anti-SARS-CoV-2 inhibitors, Cao et al. (35) synthesised and evaluated a series of GS-441524 derivatives. Among the designs, mono-isobutyryl esterification of the hydroxyl groups on the C5′ position (ATV006) showed improved activities against the Delta (EC_50_ = 0.349 μM, therapeutic index = 366.76) and Omicron (EC_50_ = 0.106 μM, therapeutic index = 1207.55) variants of SARS-CoV-2 in a Vero E6 cell model (35). In addition, ATV006 displayed excellent oral bioavailability (*F* = 81.5%), effective blood concentration (C_max_ = 8.2 μM), extensive target distribution (plasma, liver, kidneys, and lung), and potent anti-SARS-CoV-2 efficacy (reduced viral load, inflammatory cytokines, and lung damage) in mouse models (35). Notably, ATV006 and remdesivir could be metabolized to the same active triphosphate form (GS‐443902) *via* different bioconversion pathways (35, 36). ATV006 can be rapidly metabolized to GS-441524 after oral absorption, which is then intracellularly converted to monophosphate GS-441524-MP through cellular kinases and further metabolized to the bioactive triphosphate GS‐443902. In contrast, remdesivir is a monophosphorylated prodrug that does not require the first phosphorylation, which is a rate-limiting step (107). In the context of developing orally bioavailable anti-SARS-CoV-2 inhibitors, ATV006 is still in the experimental stage and has not entered clinical trials. Additional studies are thus required to demonstrate its safety, efficacy, and tolerability.

### VV116

**Figure 3:**
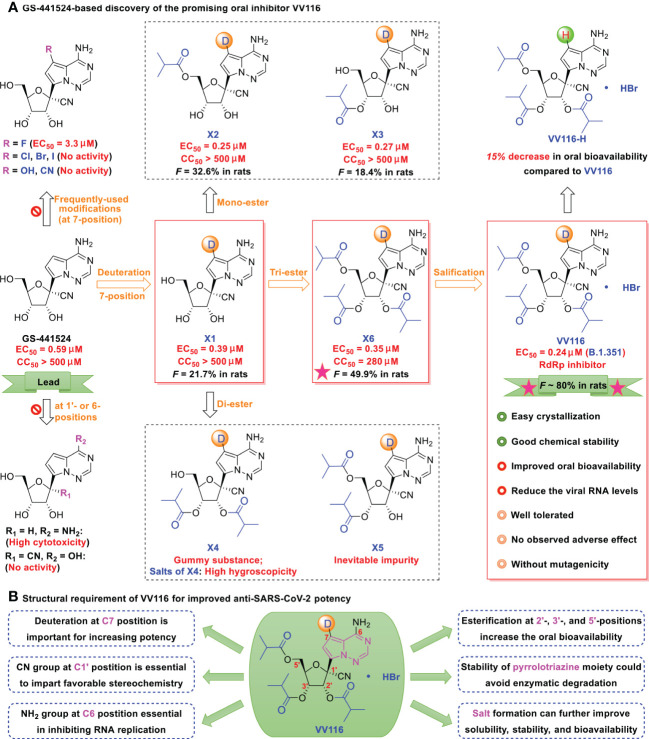
**(A)** GS-441524-based discovery of the promising oral inhibitor VV116. GS-441524 presents a promising drug candidate for further drug development. Several previous failed attempts are summarized: **(i)** introduction of frequently-used halogen (F, Cl, Br, I), hydroxyl, or cyano groups at the C7-position, resulting in less potent or no anti-SARS-CoV-2 activity, **(ii)** removal of the cyano group at C1′-position, results in high cytotoxicity, **(iii)** changing the 6-amino to a hydroxyl, methylation of the 2’-α-hydroxyl, or changing the 2’-α-hydroxyl to fluorine, results in loss of anti-SARS-CoV-2 activity. Promising attempts: deuteration at the C7 position results in strong antiviral activity; induces mono-, di- and tri-esters at the 2′-, 3′-, and 5′-positions, and improves oral bioavailability (tri-isobutyrate ester X6 [*F* ~ 50% in rats] was superior to the others in rats]); and induces hydrobromide in X6, yielding the promising oral candidate VV116 (white solid, with improved oral bioavailability, good chemical stability; no observed adverse effect, and no mutagenicity). **(B)** Structural requirement of VV116 for improved anti-SARS-CoV-2 potency.

Deuterated GS-441524 derivatives (**Figure 3**, such as X1-X6, VV116) provide a beneficial strategy for the development and selection of oral antiviral drugs. In medicinal chemistry, deuterium (D) substitution represents a valuable direction because of its potential pharmacokinetic and pharmacodynamic benefits attributed to the kinetic isotope effect (108–110). GS-441524 possesses an electron-rich pyrrolotriazine moiety that is easily oxidized by enzymes. The carbon-deuterium bond is shorter (~ 0.005 Å) than the C−H bond and is more stable under oxidative clearance processes (111, 112). Strategic deuteration could impede metabolic transformations by inhibiting oxidation or ring opening of the pyrrolotriazine moiety, leading to an increase in anti-SARS-CoV-2 activity.

As depicted in **Figure 3**, lead compound X1 was produced by deuteration and could significantly inhibit viral replication, with an EC_50_ of 0.39 μM and no observable cytotoxicity (selectivity index [SI] > 1282) (41). However, the oral bioavailability of X1 (*F* = ~ 21.7% in rats) was low because of poor solubility and liposolubility. Further esterification yielded tri-isobutyrate ester X6 (*F* = ~ 50% in rats). The detailed pharmacokinetic properties (such as maximum plasma concentration, terminal half-life, and oral bioavailability) of the related compounds are shown in **Table 1**.

To better control solubility and oral bioavailability, salification of X6 with hydrobromide proved to be a winning strategy that enhanced the oral bioavailability of VV116 (JT001, renmindevir, on a 10.8 g scale) up to 60% with respect to that of X6 in rats (41). VV116 also displayed excellent oral bioavailability in beagle dogs (*F* = 90%) and in ICR mice (*F* = 110.2%). In the mouse model, VV116 showed a dose-dependent anti-SARS-CoV-2 effect (with reduced lung injury), and high doses of VV116 markedly reduced the viral RNA copy number and improved lung histopathology (41). Overall, VV116 displays a good safety profile (high tolerated single doses: > 2.0 g/kg in rats, 1.0 g/kg in Beagle dogs) without adverse effects (14 days, 200 mg/kg in rats, 30 mg/kg in dogs), and indicates no mutagenic effects (41). Further, oral administration of VV116 showed pharmacokinetic advantages relative to its non-deuterated form VV116-H in Sprague Dawley (SD) rats (30.0 mg/kg/day, N = 3, *F* = 87.0% vs. F = 75.6%) (42).

**Figure 4:**
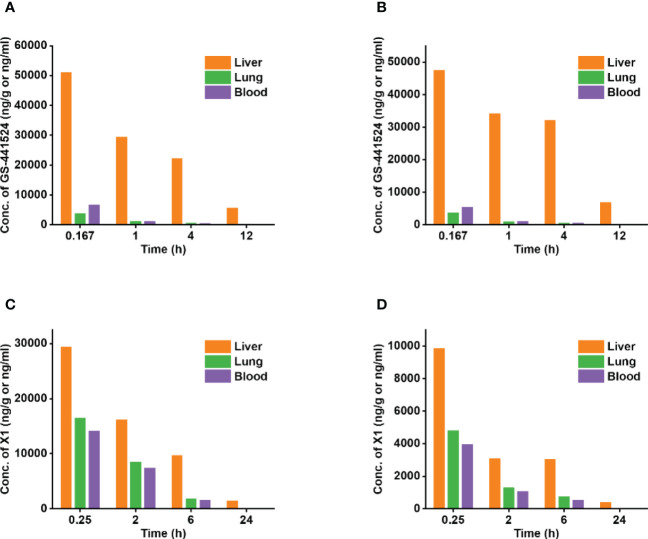
The concentration distribution of the key metabolites, GS-441524 and X1, in the liver, lungs, and blood in rats or mice. Oral VV116 administration can circumvent liver-targeting issues. **(A)** The concentration of ^[14C]^GS-441524 in the liver, lungs, and blood following intravenous administration of ^[14C]^remdesivir at a single dose of 10 mg/kg in male SD rats. **(B)** The concentration of ^[14C]^GS-441524 in the liver, lungs, and blood following intravenous administration of ^[14C]^remdesivir at a single dose of 10 mg/kg in male Long Evans rats. **(C)** The concentration of X1 in the liver, lungs, and blood following oral administration of VV116 at a single dose of 100 mg/kg in Balb/c mice. **(D)** The concentration of X1 in the liver, lungs, and blood following oral administration of VV116 at a single dose of 30 mg/kg in SD rats.

Fast-spreading SARS‐CoV‐2 variants cause resurgence of infections raise several concerns (113). However, VV116 was found to retain its anti-viral capacity against the Alpha, Beta, Gamma, Delta, as well as Omicron (EC_90_ = 0.30 μM) variants of SARS-CoV-2 (114). In terms of the molecular mechanism, VV116 functioned by targeting the highly conserved viral RdRp to block SARS-CoV-2 replication through evading “proofreading” of viral RNA sequences (41). Specifically, the postulated activation pathway of VV116 is divided into four steps: oral absorption, hydrolysis of ester group, phosphorylation (VV116-NTP), and incorporation into the growing SARS-CoV-2 RNA strand. Further, the preference of remdesivir for hepatic extraction can result in high drug accumulation and long-term toxicity (e.g., elevated liver transaminases) (114), however, the excellent tissue distribution (e.g., single oral administration in rats at 30 mg/kg) of oral VV116 can avoid these liver-targeting problems (**Figure 4**) (115).

Specifically, in male SD rats and long Evans rats groups, the liver-to-plasma concentration ratios of ^[14C]^GS-441524 (the major metabolite of ^[14C]^remdesivir) were higher than the lungs-to-plasma concentration ratios by approximately 23 times (SD rats, 1 h), 37 times (SD rats, 4 h), 34 times (Long Evans rats, 1 h), and 58 times (Long Evans rats, 4 h) (116). In contrast, the liver-to-plasma concentration ratios of 116-N1 (the major metabolite of VV116) were higher than the lungs-to-plasma concentration ratios by only 1.8 times (Balb/c mice, 0.25 h), 1.9 times (Balb/c mice, 2 h), 2.1 times (SD rats, 0.25 h), and 2.4 times (SD rats, 2 h) (116). This indicates that in contrast to the high liver-targeting capability of remdesivir, VV116 can effectively circumvent this issue.

**Figure 5:**
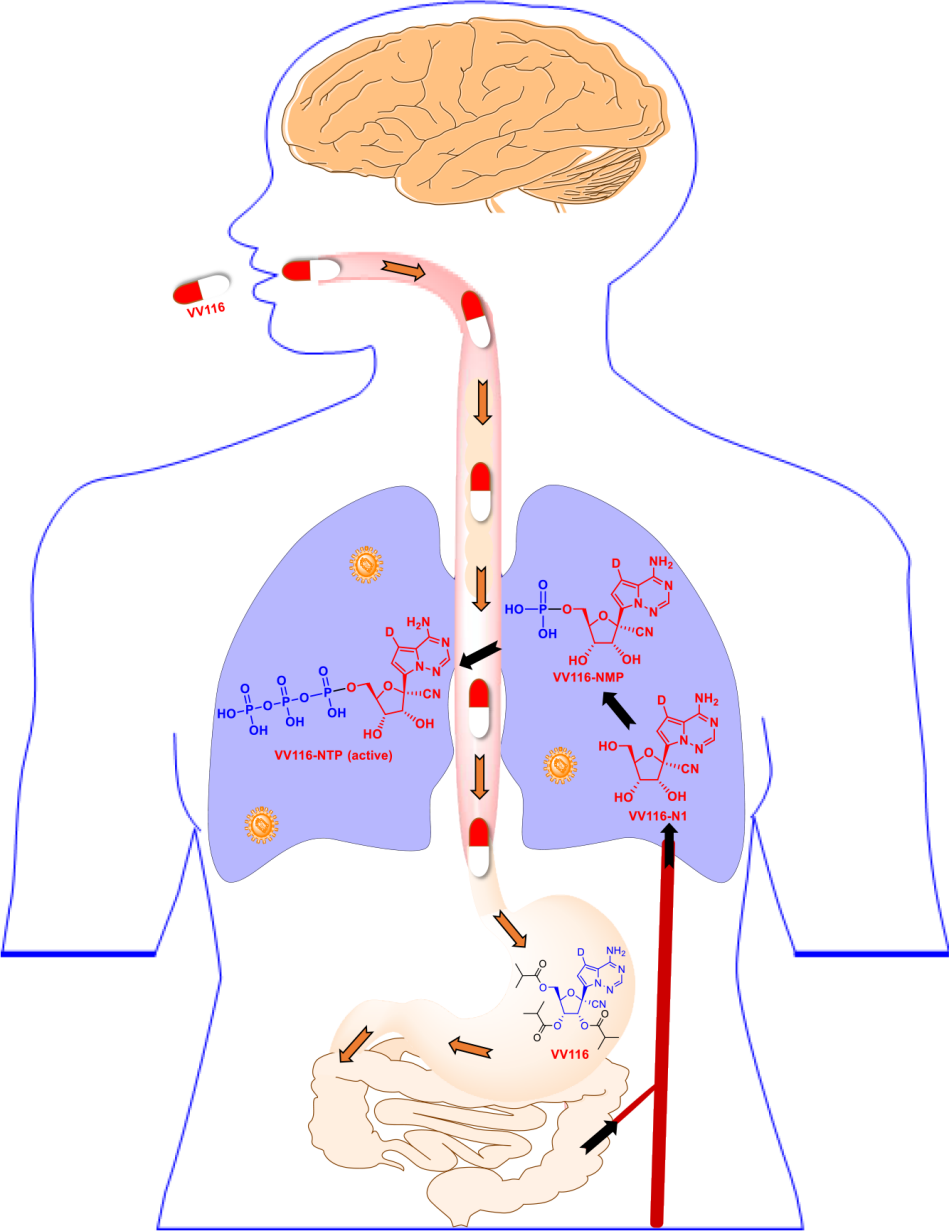
Schematic illustrating the metabolism of the oral inhibitor VV116. VV116 is an orally administered tri-isobutyrate ester prodrug of GS-441524. After intestinal absorption, VV116 is efficiently converted by cellular enzymes to the plasma metabolite VV116-N1 (median T_max_ 1.0–2.5 h), which undergoes three phosphorylation events to yield the active form, VV116-NTP.

Considering the promising therapeutic usage of VV116 against SARS-CoV-2 infection in preclinical studies, Qian et al. (43) further launched three phase I studies of VV16 in Shanghai, China (ClinicalTrials.gov: NCT05227768, NCT05201690, and NCT05221138; **Table 2**). The result found that VV116 exhibited satisfactory safety, tolerability, and pharmacokinetic properties in 86 healthy subjects (aged 18–45 years, 38 in single ascending-dose study, 36 in multiple ascending-dose study, and 12 in food-effect study). As depicted in **Figure 5**, VV116 can be efficiently converted to its active triphosphate form following oral administration. Moreover, the area under the curve (AUC) demonstrates that VV116 is quickly absorbed after the first dose with a median T_max_ of 1.00–2.50 h, and that 116-N1 is eliminated with a median t_1/2_ of 4.80–6.95 h (43).

**Table 2:** VV116 in clinical development based on a systematic search of ClinicalTrials.gov (https://clinicaltrials.gov/, accessed 31 October, 2022).

Interventions	Principal Investigator	Identifier (year)	Participants	Progress
VV116	Shanghai Xuhui Central Hospital, Shanghai, China	NCT05221138 (2021)	12	Phase I trial exhibited satisfactory safety and tolerability in Chinese healthy subjects after fasting, standard diet or high-fat diet.
VV116 (25 mg Group, 200 mg Group, 400 mg Group, 800 mg Group, 1200 mg Group)	Shanghai Xuhui Central Hospital, Shanghai, China	NCT05227768 (2021)	38	Phase I trial exhibited satisfactory safety and tolerability in Chinese healthy subjects at five dose levels.
VV116 (200 mg Group, 400 mg Group, 600 mg Group)	Shanghai Xuhui Central Hospital, Shanghai, China	NCT05201690 (2021)	36	Phase I trial exhibited satisfactory safety and tolerability in Chinese healthy subjects after multiple ascending doses.
VV116 (200 mg Group, Bid; 400 mg Group, Bid; 600 mg Group, Bid)	Nucleus Network Pty Ltd, Victoria, Australia	NCT05355077 (2022)	27	Phase I trial is in progress for Caucasian healthy subjects.
VV116	Chongqing Public Health Medical Center, Chongqing, China	NCT05242042 (2022)	1310	Phase II/III trial is in progress for the early treatment of patients with mild/moderate COVID-19, at high risk for progression to severe COVID-19, including death.
VV116 Paxlovid	Ruijin Hospital Affiliated to Shanghai Jiao Tong University School of Medicine, Shanghai, China	NCT05341609 (2022)	822	Phase III trial is in progress for the early treatment of patients with mild/moderate COVID-19.
VV116 Favipiravir	Shanghai Public Health Clinical Center, Shanghai, China	NCT05279235 (2022)	640	Phase III trial is in progress for patients with moderate/severe COVID-19.
VV116	Shanghai Vinnerna Biosciences Co., Ltd., Shanghai, China	NCT05582629 (2022)	1200	Phase III trial is in progress for patients with mild/moderate COVID-19

Furthermore, Shen et al. (114) conducted an open, prospective cohort study in China, including 136 hospitalised patients with non-severe COVID-19 caused by the Omicron variant, 60 of whom patients received VV116 (300 mg, twice daily for 5 days), and found that the viral shedding time of the VV116 group was significantly shorter than that of the control group (8.56 *vs.* 11.13 days). Nine mild adverse events occurred in the VV116 group, and all resolved without required intervention (114). VV116 targets highly conserved RdRp (only one mutation in nsp12, distant from the RdRp active site), which may explain why it maintains high potency against the Omicron variant (117). Moreover, VV116 has been further investigated in five clinical trials (ClinicalTrials.gov: NCT05242042, NCT05279235, NCT05341609, NCT05355077, and NCT05582629; As depicted in **Table 2**), and their findings will be disclosed shortly.

### GS-621763

Cox et al. (40) demonstrated that GS-621763 (X6-H; white solid), which is generated by tri-isobutyryl esterification of the hydroxyl groups of GS-441524 at the C5′, C2′, and C3′ positions, represents another example of a GS-441524 prodrug with enhanced oral bioavailability, which could significantly reduce the SARS-CoV-2 burden to near-undetectable levels in ferrets infected with the gamma variant. SARS-CoV-2 infection can have long-term effects on pulmonary and multiple extrapulmonary tissues and organs (118). Schäfer et al. (119) showed that oral delivery of GS-621763 could diminish SARS-CoV-2 replication, improve pulmonary function, and prevent COVID-19 progression in BALB/c model mice. The potential anti-SARS-CoV-2 had been preliminarily revealed, while the safety, efficacy, and pharmacokinetic properties of GS-621763 are still an open question and require additional studies.

Although the potential anti-SARS-CoV-2 had been preliminarily revealed, at the same time, we must understand that oral GS-441524 derivatives (VV116, ATV006, and GS-621763) have not been deeply investigated yet. The clinical data are still limited (only VV116 has entered in clinical development, as shown in (**Table 2**), we call for more comprehensive studies.

## Conclusion and discussion

Viral RdRp is a valuable target for COVID-19 therapeutic interventions because it is highly conserved among SARS-CoV-2 variants. Oral administration has the potential to maximize clinical benefits, including decreased duration of COVID-19 and reduced post-acute sequelae of SARS-CoV-2 infection, whereas remdesivir (version 1.0 of GS-441524) is only suitable for injection. The currently available data support the exploration of next-generation oral inhibitors of SARS-CoV-2 polymerase (version 2.0, GS-441524) for treating COVID-19. This exploration could enhance preparedness for future outbreaks of SARS-CoV-2 and improve therapeutic efficacy during the current pandemic. Oral GS-441524 derivatives, including VV116, ATV006, and GS-621763, have potential as the cornerstone of first-line defence against COVID-19.

Specifically, a promising oral version of GS-441524 should have the following key characteristics: (i) enhanced oral bioavailability, tissue-specific localization, and plasma half-life; (ii) sufficient safety (excellent CC_50_) and resistance profile; (iii) significantly reduced viral loads and dramatically decreased lung injury; (iv) maximally maintained antiviral activity against SARS-CoV-2 variants; and (v) large-scale manufacturing ability for increased accessibility and affordability to outpatients (VV116 is easier to synthesize than remdesivir).

Although the potential oral anti-SARS-CoV-2 agents have been preliminarily revealed, the relevant data remain limited, because only VV116 has reached the clinical stage (**Table 2**). Considering the positive effects of oral GS-441524 derivatives on COVID-19 and the urgent need to explore possible SARS-CoV-2 treatments, it is crucial to systematically elucidate their safety, efficacy, tolerability, and pharmacokinetic properties through large-scale preclinical and clinical studies. Notably, GS-441524 showed high interpatient variability towing to differences in renal function (**Table 1**) (39), implying that the related pharmacokinetics (including potential efficacy and toxicity) of oral GS-441524 derivatives need to be investigated further to rationally evaluate the possibility of its clinical application and to further guide drug development.

Meanwhile, multiple measures can be considered. Deuterated GS-441524 derivatives provide a beneficial strategy for the development and selection of oral antiviral drugs. Deuteration has gained overwhelming popularity (120). However, owing to synthetic difficulties, only 7-deuterated derivatives of GS-441524 (pyrrolotriazine moiety) have been obtained. Additional site-specific deuterium substitution derivatives need to be synthesized through novel efficient synthetic reactions to exert kinetic isotope effects, enhance oral bioavailability, and exploit SARS-CoV-2-specific antiviral drugs with clinical advantages. Further, optimized drug combinations (such as VV116 + PF‐07321332, VV116 + EIDD-2801, VV116 + masitinib, and VV116 + F0213) that target multiple routes can both enhance synergistic efficacy and reduce drug resistance and toxicity (121–125). However, any potential combination must verify the anticipated synergistic/additive effects and drug-drug interactions through systematic studies. Further, a VV116-based nano delivery system (with enhanced bioavailability, precision, and sustained drug release) can be a good therapeutic alternative for SARS-CoV-2 infection. However, as the relevant studies are limited, this approach should considered and elucidated further.

With continuing advances, more effective oral GS-441524 derivatives can be developed. However, to date, VV116 is the top contender for clinical development owing to its large-scale manufacturing ability, excellent oral bioavailability and safety profile, reduced viral RNA copies, and improved lung histopathology.

## Author contributions

ZW: Conceptualization, collecting the literatures, writing-original draft, writing-review & editing, visualization, funding acquisition. LY: Conceptualization, writing-review & editing, funding acquisition. X-QS: Collecting the literatures, editing, visualization. All authors contributed to the article and approved the submitted version.
